# Accessible New Non-Quantum Dot Cs_2_PbI_2_Cl_2_-Based Photocatalysts for Efficient Hole-Driven Photocatalytic Applications

**DOI:** 10.3390/molecules29143249

**Published:** 2024-07-09

**Authors:** Xing Huang, Kuanxin Lv, Wenqiang Zhu, Zhenzhen Li, Hang Zhao

**Affiliations:** College of Metallurgy and Energy, North China University of Science and Technology, Tangshan 063210, China; minthx@163.com (X.H.); 15148112525@163.com (K.L.); zwq9788@163.com (W.Z.)

**Keywords:** perovskite photocatalysis, two-dimensional Cs_2_PbI_2_Cl_2_-based materials, hole-driven photocatalytic process, stable, high-efficient photocatalysts

## Abstract

Efficient, low-cost photocatalysts with mild synthesis conditions and stable photocatalytic behavior have always been the focus in the field of photocatalysis. This study proves that non-quantum-dot Cs_2_PbI_2_Cl_2_-based materials, created by a simple method, can be successfully employed as new high-efficient photocatalysts. The results demonstrate that two-dimensional Cs_2_PbI_2_Cl_2_ perovskite can achieve over three times higher photocatalytic performance compared to three-dimensional CsPbBr_3_ perovskite. Moreover, the photocatalytic performance of Cs_2_PbI_2_Cl_2_ can be further improved by constructing a heterojunction structure, such as Cs_2_PbI_2_Cl_2_/CsPbBr_3_. Cs_2_PbI_2_Cl_2_ can connect well with CsPbBr_3_ through a simple method, resulting in tight bonding at the interface and efficient carrier transfer. Cs_2_PbI_2_Cl_2_/CsPbBr_3_ exhibits notable 5-fold and 10-fold improvements in photocatalytic performance and rate compared to CsPbBr_3_. Additionally, Cs_2_PbI_2_Cl_2_/CsPbBr_3_ demonstrates superb stable catalytic performance, with nearly no decrease in photocatalytic performance after 7 months (RH = 20% ± 10, T = 25 °C ± 5). This study also reveals that the photocatalytic process based on Cs_2_PbI_2_Cl_2_/CsPbBr_3_ can directly oxidize organic matter using holes, without relying on the generation of intermediate reactive oxygen species from water or oxygen (such as ·OH or ·O_2_^−^), showcasing further potential for achieving high photocatalytic efficiency and selectivity in anhydrous/anaerobic catalytic reactions and treating recalcitrant pollutants.

## 1. Introduction

Currently, non-metallic and metal oxide (sulfide) semiconductor materials are selected as the most commonly used photocatalysts in the photocatalysis field [1,2]. For these photocatalysts, a high-temperature annealing process is usually required, and the wide band gap for the most efficient oxide materials only allows them to be effectively used in the ultraviolet light range [3]. In addition, proper control of the nanoparticle size is also necessary. For example, the titanium dioxide (TiO_2_) particles should be controlled at the 10–30 nm level to ensure a high specific surface area for achieving high photocatalytic performance [4]. Moreover, superoxide anions or hydroxyl intermediate radicals are usually required to ensure the smooth progress of the photocatalytic process driven by these oxide catalysts, the performance of which often depends on the level of dissolved oxygen concentration or water molecules [5]. In the photocatalysis field, the development of new photocatalysts with a high efficiency, low cost, easy synthesis, non-dependence of intermediate active radicals, and stable photocatalytic behavior has always been a focus of photocatalytic studies [6].

Due to their outstanding photophysical characteristics, perovskite materials have emerged as an effective light-absorbing layer for solar cells, and their solar cells can achieve a photoelectric conversion efficiency of over 26% [7]. As suitable candidates for efficient photocatalysts, the prime characteristics of photo-generated carriers and the transmission of carriers are effective [8,9]. Among perovskites, the cesium lead bromide (CsPbBr_3_) perovskite with the suitable tolerance factor of 0.82 is well maintained as the perovskite structure [10,11,12,13]. Recently, some attempts have been made to use CsPbBr_3_ nanocrystals in the form of quantum dots as different kinds of photocatalysts. During organic reactions, the CsPbBr_3_ quantum dot, when used as a catalyst, has been identified to accelerate bond formations, with a high yield [14]. As a useful organic pollutant degradation photocatalyst, the CsPbBr_3_ quantum dot can effectively degrade the organic compound of 2-Mercaptobenzothiazole [15]. Additionally, the construction of CsPbBr_3_ -based heterostructures, such as CsPbBr_3_/TiO_2_, has been adopted to reduce the nonradiative recombination of carriers, ultimately improving the photocatalytic performance of CsPbBr_3_ [16,17].

To date, studies on CsPbBr_3_ or its heterostructure photocatalysts mainly focus on the CsPbBr_3_ quantum dot due to its improved stability and quantum size effect [18,19,20,21,22]. Nevertheless, the preparation of quantum dots often involves issues with the cost of preparation and controlling the yield of the high-quantity nanocrystals [23,24,25,26]. Additionally, most heterojunction-structured CsPbBr_3_ photocatalysts are still involved with a high-temperature preparation process (>300 °C) due to high-temperature-annealed materials, such as TiO_2_ [27]. Meanwhile, there are some problems, such as the fact that the surface ligands of quantum dots, such as oleyl amine and oleic acid, can eliminate the holes contributing to the photocatalytic processes, or unwanted or uncontrolled photoreactions can occur, ultimately leading to deceased perovskite stability and limited photocatalytic performance [28]. To face these issues, it is valuable to further explore new-structured and new-type perovskites for photocatalytic applications.

In this study, non-quantum-dot, Cs_2_PbI_2_Cl_2_-based perovskite materials were investigated as new-type photocatalysts for efficient hole-driven photocatalytic applications. The results indicate that these photocatalysts can be easily obtained at low temperatures (140 °C) by a simple solution method. Compared with CsPbBr_3_, Cs_2_PbI_2_Cl_2_ photocatalysts can achieve 3-fold and 7-fold improvements in photocatalytic performance and rate. Moreover, by constructing the Cs_2_PbI_2_Cl_2_/CsPbBr_3_ heterostructure structure, due to the tight bonding of interface and suitable valence band, the photocatalytic performance and rate can be further increased by 5 times and 10 times. Additionally, Cs_2_PbI_2_Cl_2_/CsPbBr_3_ photocatalysts show excellent photocatalytic stability, with almost no decrease in photocatalytic performance after 7 months in the air and after cyclic catalytic tests. Additionally, the results also reveal that the photocatalytic process based on Cs_2_PbI_2_Cl_2_/CsPbBr_3_ can be directly driven by holes, without relying on the generation of intermediate reactive oxygen species generated from water or oxygen (such as ·OH or ·O_2_^−^), which exhibit further potential for achieving the high photocatalytic efficiency and selectivity.

## 2. Results and Discussion

**Figure 1:**
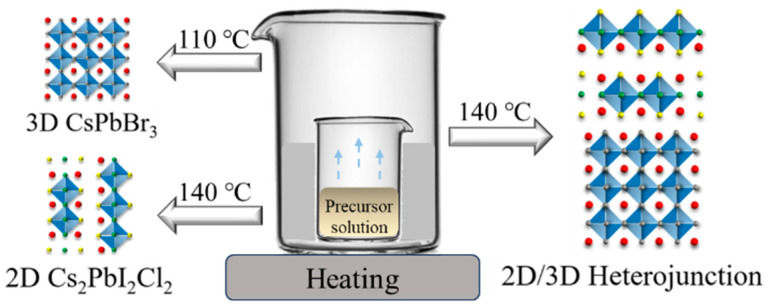
The schematic of preparation for CsPbBr_3_, Cs_2_PbI_2_Cl_2_, and mixed-dimensional Cs_2_PbI_2_Cl_2_/CsPbBr_3_ perovskite photocatalyst.

In this study, perovskite photocatalysts were synthesized by a simple solution evaporation method. The details of these processes are shown in the Supplementary Materials. The schematic of preparation for CsPbBr_3_, Cs_2_PbI_2_Cl_2_ and Cs_2_PbI_2_Cl_2_/CsPbBr_3_ perovskite photocatalysts is shown in Figure 1. Taking Cs_2_PbI_2_Cl_2_/CsPbBr_3_ as an example, CsCl and PbI_2_ (molar ratio of 2:1) are added to dimethyl sulfoxide (DMSO) to prepare the solution, and the pre-prepared non-quantum-dot CsPbBr_3_ crystals are subsequently dropped into the solution. The Cs_2_PbI_2_Cl_2_/CsPbBr_3_ photocatalyst can be created by evaporating the solution at a temperature of 140 °C.

**Figure 2:**
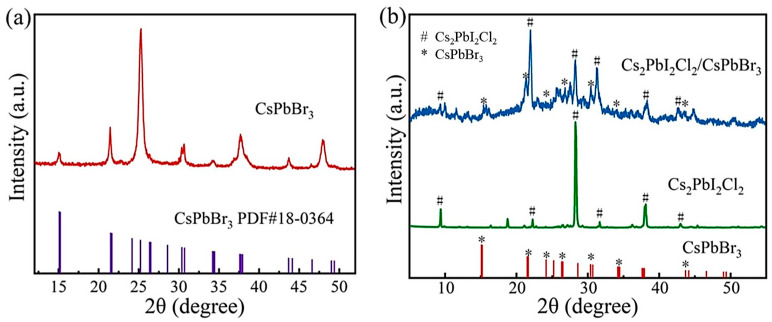
(**a**,**b**) The XRD patterns of CsPbBr_3_, Cs_2_PbI_2_Cl_2_, and Cs_2_PbI_2_Cl_2_/CsPbBr_3_ crystals.

The XRD measurement was used to detect the crystal structure obtained by the solution evaporation method. As shown in Figure 2a, the diffraction peaks at 2θ = 15.21°, 21.49°, 26.34°, 30.37°, and 34.19° corresponded to the (001), (110), (111), (002), and (210) crystal planes of CsPbBr_3_ perovskite, comprising the standard cards of CsPbBr_3_ perovskite (PDF # 18-036). The XRD patterns of Cs_2_PbI_2_Cl_2_ and mixed-dimensional Cs_2_PbI_2_Cl_2_/CsPbBr_3_ perovskite are shown in Figure 2b. The diffraction peaks of XRD patterns at 2θ = 9.07°, 21.77°, 27.60°, and 32.35° correspond to the (002), (110), (105), and (202) crystal planes of Cs_2_PbI_2_Cl_2_, which is consistent with our early research. The XRD pattern of mixed compounds is shown in Figure 2b. As demonstrated by the comparison of XRD patterns in Figure S1, the characteristic diffraction peaks representative of Cs_2_PbI_2_Cl_2_ and CsPbBr_3_ in the Cs_2_PbI_2_Cl_2_/CsPbBr_3_ compounds shift towards lower and higher angles, respectively. This indicates that the partial substitution of Cl in CsPbBr_3_ and Br in Cs_2_PbI_2_Cl_2_ may have occurred during the synthesis of Cs_2_PbI_2_Cl_2_/CsPbBr_3_ compounds.

**Figure 3:**
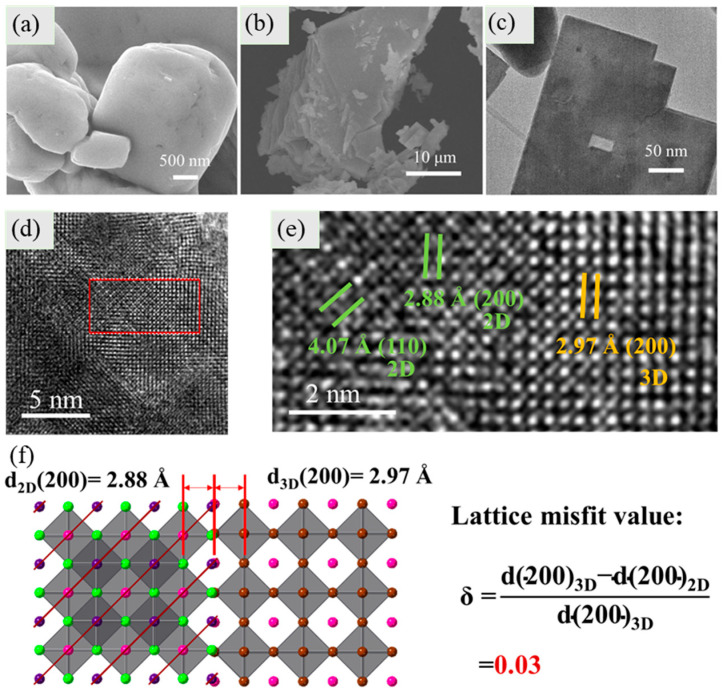
SEM images of (**a**) CsPbBr_3_, (**b**) Cs_2_PbI_2_Cl_2_, and (**c**) Cs_2_PbI_2_Cl_2_/CsPbBr_3_ catalysts; (**d**) HRTEM results of Cs_2_PbI_2_Cl_2_/CsPbBr_3_ photocatalyst crystals; (**e**) The partial area enlarged pattern extracted from (**d**); and (**f**) schematic diagram of crystal structure arrangement of Cs_2_PbI_2_Cl_2_/CsPbBr_3_ and the lattice compatibility.

To observe the CsPbBr_3_, Cs_2_PbI_2_Cl_2_, and mixed-dimensional Cs_2_PbI_2_Cl_2_/CsPbBr_3_ crystals, scanning electron microscopy (SEM) was performed. It can be observed that the CsPbBr_3_ crystals have a bulk shape (Figure 3a), and the Cs_2_PbI_2_Cl_2_ crystals display a sheet-like accumulation (Figure 3b). After combining the two types of crystals, it was detected that the crystals had a clear sheet-like shape, but the CsPbBr_3_ crystals were not clearly observed (Figure 3c). To further identify the combination of two crystals, high-resolution transmission electron microscopy (HRTEM) was used. As observed from the results (Figure S2a,b), it can be seen that the Cs_2_PbI_2_Cl_2_/CsPbBr_3_ crystals have a platelike shape, and the dark spots are distributed on the plane of platelike crystals. The lattice fringes observed from the microregion of Cs_2_PbI_2_Cl_2_/CsPbBr_3_ crystal (Figure 3d) displayed interplanar d-spacings of 2.88 Å and 4.07 Å (Figure 3e), which was consistent with the lattice parameters of the (200) and (110) planes for the two-dimensional Cs_2_PbI_2_Cl_2_ crystal. Meanwhile, the interplanar d-spacings of 2.97 Å for the (200) plane of the CsPbBr_3_ crystal were also observed from the TEM pattern shown on the right side of Figure 3e. The lattice misfit values of (200) of Cs_2_PbI_2_Cl_2_ and (200) of CsPbBr_3_ were calculated using δ = (d(200)_2D_-d(200)_3D_)/d(200)_3D_ (Figure 3f). The lattice misfit value was calculated as 3% (<5%), which conveniently formed the coherent interface between the different crystal structures according to the semi-coherent dislocation theory [29,30]. The small difference in the d-spacings of two crystals implies the small interfacial energy between (200) of Cs_2_PbI_2_Cl_2_ and (200) of CsPbBr_3_, which is beneficial for their connection. As a result, the interface of Cs_2_PbI_2_Cl_2_ and CsPbBr_3_ structures showed a tight connection, and the lattice structures displayed a smooth transition from the (200) of Cs_2_PbI_2_Cl_2_ to (200) of CsPbBr_3_ (Figure 3e,f). Such a tight connection and smooth transition at the interface between Cs_2_PbI_2_Cl_2_ and CsPbBr_3_ would effectively reduce the interface carrier transport barrier and facilitate the smooth transmission of photocarriers, thereby reducing the probability of non-radiative recombination and helping to achieve a high photocatalytic performance. As evidenced by XRD and HRTEM results, the Cs_2_PbI_2_Cl_2_/CsPbBr_3_ mixed-dimensional heterojunction crystal was successfully obtained through a simple solution process.

**Figure 4:**
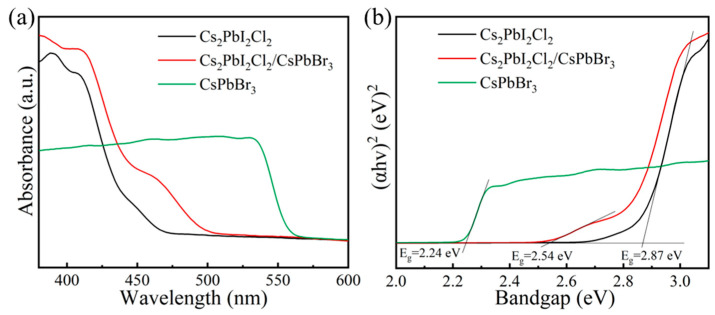
(**a**) UV–Vis and (**b**) UV–Vis DRS results of CsPbBr_3_, Cs_2_PbI_2_Cl_2_, and Cs_2_PbI_2_Cl_2_/CsPbBr_3_ photocatalysts.

The optical absorption range and bandgap widths of the CsPbBr_3_, Cs_2_PbI_2_Cl_2_ and Cs_2_PbI_2_Cl_2_/CsPbBr_3_ heterojunction crystalline materials were assessed using ultraviolet-visible absorption spectroscopy (UV–Vis) and ultraviolet–visible diffuse reflectance spectroscopy (UV–Vis DRS). The UV–Vis results revealed that the absorption cutoff edge of CsPbBr_3_ at approximately 560 nm, while the presence of CsPbBr_3_ extends the cutoff edge of Cs_2_PbI_2_Cl_2_/CsPbBr_3_ from 460 nm (Cs_2_PbI_2_Cl_2_) to around 500 nm (Figure 4a). By fitting curves using the Tauc Plot method in the UV–Vis DRS test, the bandgaps of CsPbBr_3_, Cs_2_PbI_2_Cl_2_, and Cs_2_PbI_2_Cl_2_/CsPbBr_3_ crystalline materials were determined to be 2.24 eV, 2.87 eV, and 2.54 eV, respectively (Figure 4b). 

**Figure 5:**
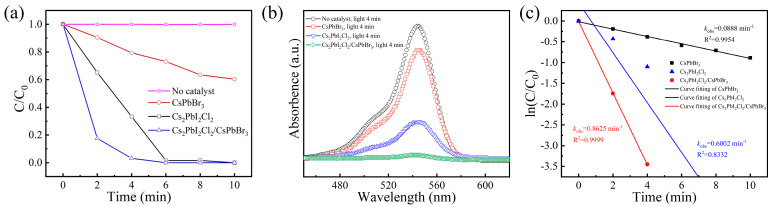
(**a**) Photocatalytic performance tests of CsPbBr_3_, Cs_2_PbI_2_Cl_2_, and Cs_2_PbI_2_Cl_2_/CsPbBr_3_ photocatalysts; (**b**) UV−Vis absorption of organic solution without and with catalysis by CsPbBr_3_, Cs_2_PbI_2_Cl_2_, and Cs_2_PbI_2_Cl_2_/CsPbBr_3_ after 4 min; (**c**) reaction rates of CsPbBr_3_, Cs_2_PbI_2_Cl_2_, and Cs_2_PbI_2_Cl_2_/CsPbBr_3_ fitted by using first−order kinetics.

To evaluate the photocatalytic performance of the CsPbBr_3_, Cs_2_PbI_2_Cl_2_ and Cs_2_PbI_2_Cl_2_/CsPbBr_3_ as photocatalysts, the representative organic pollutant Rhodamine B was selected as the standard material. Additionally, the change in the absorbance of the Rhodamine B solution under the simulated sunlight illumination (AM 1.5 G) was used to judge the photocatalytic performances (Figure 5a, where C_0_, A_0_, C, and A represent the concentration and absorbance before and after degradation, respectively). The results of sampling every 2 min displayed that, without any photocatalyst, the organic matter showed no decomposition after 10 min under the light exposure. For the CsPbBr_3_-catalyzed case, only a ~10% and ~20% degradation of organic matter occurred during the relatively high catalytic rate stage after 2 and 4 min. In contrast, the Cs_2_PbI_2_Cl_2_ catalyst degraded 36.4 and 67.8% of the organic matter within the same time, which showed that the improvement in performance was at least triple. Impressively, Cs_2_PbI_2_Cl_2_/CsPbBr_3_ degraded 81.8% and 97.3% of the organic matter after 2 and 4 min, and all organic matter was degraded within 6 min. Although the UV–Vis results showed that Cs_2_PbI_2_Cl_2_ and Cs_2_PbI_2_Cl_2_/CsPbBr_3_ possessed a narrower light absorption range compared to CsPbBr_3_, they could exhibit an improvement in photocatalytic performance of 3–8 times.

This enhancement in performance could also be deduced from the UV–Vis results after 4 min (Figure 5b). The solution catalyzed by CsPbBr_3_ exhibited strong absorption peaks of organic matter, while the Cs_2_PbI_2_Cl_2_-based solution showed an evident decrease in absorption peaks, and the Cs_2_PbI_2_Cl_2_/CsPbBr_3_-based solution showed very weak absorption peaks. Furthermore, the degradation rates of the samples were simulated using first-order kinetics, as shown in Figure 5c. According to the simulating results, the degradation rate of CsPbBr_3_ was 0.089 min^−1^. In contrast, the degradation rates for Cs_2_PbI_2_Cl_2_ and the Cs_2_PbI_2_Cl_2_/CsPbBr_3_ heterojunction structure could reach 0.600 min^−1^ and 0.863 min^−1^, exhibiting almost a 7–10 times higher photocatalytic rate. In order to better estimate Cs_2_PbI_2_Cl_2_-based photocatalytic performance, the TiO_2_ with a 20 nm particle size possessing a relatively high catalytic performance and CsPbBr_3_ were selected to construct the heterojunction photocatalyst for comparison (Figure S4). It can be observed that within 4 min, 35% of the organic matter was not catalyzed by the TiO_2_/CsPbBr_3_ photocatalyst, and all the organic matter could not be completely catalyzed by TiO_2_/CsPbBr_3_ after 10 min. Meanwhile, all Rhodamine B was degraded by the Cs_2_PbI_2_Cl_2_-based photocatalysts within 6–10 min, which implied a high-efficient photocatalytic performance of Cs_2_PbI_2_Cl_2_-based photocatalysts, especially for the Cs_2_PbI_2_Cl_2_/CsPbBr_3_ heterojunction photocatalyst.

**Figure 6:**
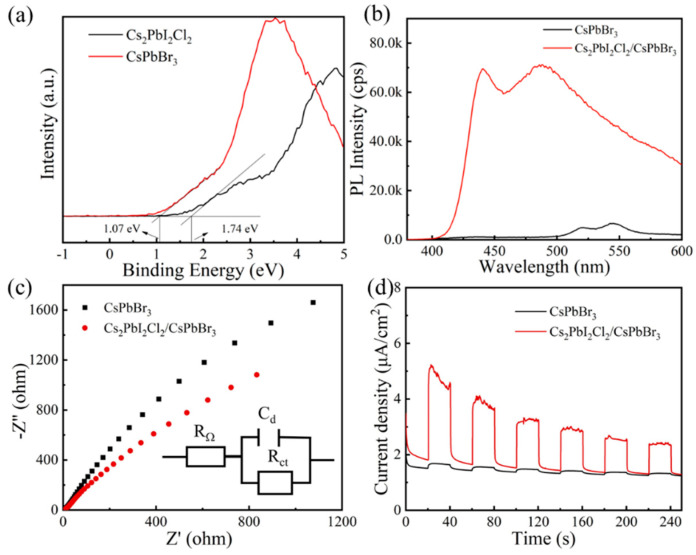
(**a**) XPS, (**b**) PL, (**c**) EIS and (**d**) transient photocurrent response measurements of CsPbBr_3_ and Cs_2_PbI_2_Cl_2_ photocatalysts.

Furthermore, the photogenerated carrier transfer characteristics of the Cs_2_PbI_2_Cl_2_/CsPbBr_3_ heterojunction crystals were explored through X-ray photoelectron spectroscopy (XPS) and steady-state photoluminescence (PL) measurement. The XPS measurements (Figure 6a) show that the valence band maximum (EVB) for Cs_2_PbI_2_Cl_2_ and CsPbBr_3_ are at 1.84 eV and 1.07 eV, which suggests that a suitable band offset exists between Cs_2_PbI_2_Cl_2_ and CsPbBr_3_ perovskite. As observed from PL measurements (Figure 6b), compared to the fluorescence peak of CsPbBr_3_, the peak of the Cs_2_PbI_2_Cl_2_/CsPbBr_3_ heterojunction exhibited a significant increase in fluorescence intensity. This means that the non-radiative recombination of photogenerated carriers in Cs_2_PbI_2_Cl_2_/CsPbBr_3_ were more effectively reduced, which was beneficial for providing more active electrons and holes to improve photocatalytic efficiency.

To identify carrier mobility, electrochemical impedance spectroscopy (EIS) tests were performed. The results show that the Cs_2_PbI_2_Cl_2_/CsPbBr_3_ powder exhibited a smaller radius of curvature compared to CsPbBr_3_ powder (Figure 6c) and TiO_2_/CsPbBr_3_ powder (Figure S5a). This indicates that the Cs_2_PbI_2_Cl_2_/CsPbBr_3_ powder heterojunction material possesses lower carrier transfer resistance, facilitating more rapid and efficient carrier transfer. The results from transient photocurrent response tests demonstrated that the Cs_2_PbI_2_Cl_2_/CsPbBr_3_ heterojunction could generate a higher current density than that of CsPbBr_3_ (Figure 6d) and TiO_2_/CsPbBr_3_ (Figure S5b), further proving the advantage of Cs_2_PbI_2_Cl_2_/CsPbBr_3_ heterostructure in carrier transfer and transport. When analyzing our previous research, the high photocatalytic performance of Cs_2_PbI_2_Cl_2_/CsPbBr_3_ should be attributed to the reduced non-radiative recombination, the tight connection of heterojunction structure interface (Figure 3), and the matched energy level (Figure 6a). The rapid and efficient carrier transfer will reduce the probability of non-radiative recombination of holes and electrons, thereby facilitating the participation of more photo-generated active electrons and holes in accelerating photocatalytic processes.

**Figure 7:**
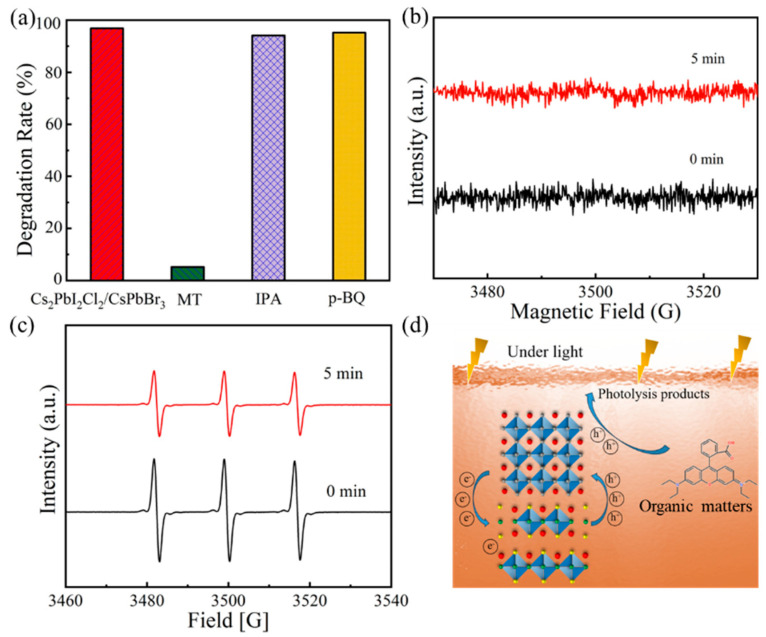
(**a**) Free radical capture tests for Cs_2_PbI_2_Cl_2_ photocatalysts; (**b**,**c**) ESR for ·O_2_^−^ generation and h^+^ generation for Cs_2_PbI_2_Cl_2_ photocatalysts; and (**d**) schematic diagram of Cs_2_PbI_2_Cl_2_ photocatalytic mechanism of organic matters.

To further our understanding of the photocatalytic mechanisms generated by the Cs_2_PbI_2_Cl_2_/CsPbBr_3_, free radical capture experiments were conducted (Figure 7a). Methanol (MT), isopropanol (IPA), and p-benzoquinone (p-BQ) were adopted as the holes (h^+^), and hydroxyl radicals (·OH) and superoxide radicals (·O_2_^−^) were adopted as free radical scavengers, which usually participate in the photocatalytic process [31,32]. According to the results, it could be seen that after 4 min, the degradation rates of organic matter were decreased from 97.3% to 93.8% and 94.4%, respectively, after the addition of IPA and p-BQ scavengers, which showed no significant effect on the degradation processes. This indicated that ·OH and ·O_2_^−^ were not the primary active species in the photocatalytic reaction driven by Cs_2_PbI_2_Cl_2_/CsPbBr_3_. When MT was added, the degradation rate was significantly decreased to 5.2%, successfully inhibiting the degradation process. Therefore, in the photocatalytic process driven by Cs_2_PbI_2_Cl_2_/CsPbBr_3_, the h^+^ should play the crucial role in the degradation of organic matters.

Moreover, electron spin resonance (ESR) tests were further performed to validate the crucial role of h^+^ and ·O_2_^−^ formed by oxygen/electronics during the photocatalytic processes driven by Cs_2_PbI_2_Cl_2_/CsPbBr_3_. In the electron spin resonance detection of ·O_2_^−^ generated from electronics, as shown in Figure 7b, no ESR fluctuation signal appeared under dark conditions and after 5 min of illumination, suggesting that ·O_2_^−^ was indeed not an active radical species participating in the photocatalytic process. This means that the dissolved oxygen in solution is not an essential requirement to ensure that ·O_2_^−^ drives the Cs_2_PbI_2_Cl_2_/CsPbBr_3_-based photocatalytic process. To validate the role of photogenerated h^+^, the 2,2,6,6-tetramethylpiperidinooxy (TEMPO) additive was chosen as the capture agent (Figure 7c). As evidenced by the results, under the dark conditions, a high signal peak of TEMPO was detected. When the Cs_2_PbI_2_Cl_2_/CsPbBr_3_ heterojunction material generated h^+^ under illumination for 5 min, the h^+^ neutralized with TEMPO, resulting in a significant decrease in the TEMPO signal. These results indicate that the generation of h^+^ is the main factor affecting the photocatalytic reaction driven by Cs_2_PbI_2_Cl_2_/CsPbBr_3_ (Figure 7d). Hence, the dissolved oxygen and water molecules are not necessary to form the ·O_2_^−^ and ·OH intermediate active radicals that guarantee the proceeding of the photocatalytic process. For the hole-driven photocatalytic processes of Cs_2_PbI_2_Cl_2_/CsPbBr_3_, it can provide a high-efficient photocatalytic performance due to avoiding energy loss, benefiting from the absence of the need to form ·O_2_^−^ and ·OH intermediate radicals. Meanwhile, the hole-direct-driven catalytic process of Cs_2_PbI_2_Cl_2_-based photocatalysts usually has a high oxidation capacity and exhibits a reduction in side reactions, which are very suitable for treating recalcitrant pollutants or the catalytic applications of anhydrous and anaerobic organic chemical reactions.

**Figure 8:**
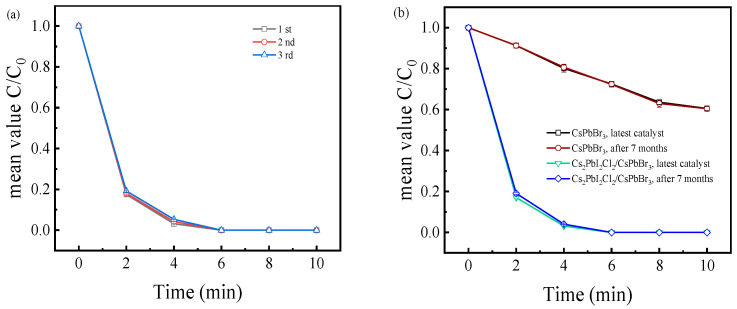
(**a**) The cyclic recirculation catalytic performance and (**b**) environmental stability tests for Cs_2_PbI_2_Cl_2_/CsPbBr_3_ catalysts.

Furthermore, the Cs_2_PbI_2_Cl_2_/CsPbBr_3_ photocatalyst was proven to possess an excellent photocatalytic stability. The performance stability of the Cs_2_PbI_2_Cl_2_/CsPbBr_3_ photocatalyst was evaluated through the cyclic catalytic performance and environmental stability tests. The results of the cyclic tests showed that after three cycles, the performance of the Cs_2_PbI_2_Cl_2_/CsPbBr_3_ photocatalyst only exhibited minor changes within the time period of 2 to 6 min, but the entire catalytic process was still completed within 6 min (Figure 8a). Meanwhile, attributed to the template stabilization effect of stable two-dimensional Cs_2_PbI_2_Cl_2_ perovskite structured on three-dimensional CsPbBr_3_ structures, the catalytic performance showed almost no change after the catalyst was placed in normal storage environment (RH = 20% ± 10, T = 25 °C ± 5) for 7 mouths (>5000 h) (Figure 8b). This shows that durable high-catalytic-efficiency characteristics are more suitable for the application of organic chemical catalytic reactions. To further identify the stability, accelerated aging tests were performed (RH= 60%) (Figure S6). After 3 months, a notable attenuation occurred in the reaction rate of CsPbBr_3_-based photocatalytic processes (Figure S6), which decreased from 0.089 min^−1^ to 0.045 min^−1^ (attenuation rate: 49.4%; Figure S7). The stability of Cs_2_PbI_2_Cl_2_/CsPbBr_3_ exhibited a significant improvement. The reaction rate of Cs_2_PbI_2_Cl_2_/CsPbBr_3_-based photocatalytic processes decreases from 0.863 to 0.669 (attenuation rate: 22.4%; Figure S7), and Cs_2_PbI_2_Cl_2_/CsPbBr_3_ could still complete all of the catalytic reaction within 6–8 min (Figure S6).

## 3. Conclusions

In conclusion, the non-quantum-dot, Cs_2_PbI_2_Cl_2_-based perovskites created using our efficient method are proven to be new-type high-efficient photocatalysts. The catalytic performance of Cs_2_PbI_2_Cl_2_ is three times better than that of the single CsPbBr_3_ photocatalyst, and its catalytic rate is seven times higher. The results confirm that the two-dimensional Cs_2_PbI_2_Cl_2_ and CsPbBr_3_ may be effectively coupled to create a tightly connected and smoothly transitioning heterojunction structure using a simple method. The tight bonding of the interface and suitable valence band maximum of Cs_2_PbI_2_Cl_2_/CsPbBr_3_ result in the efficient transfer of carriers and the promotion of photocatalytic process. The photocatalytic performance can be enhanced even further, resulting in a 5-fold and 10-fold increase in catalytic performance and rate. Moreover, Cs_2_PbI_2_Cl_2_/CsPbBr_3_ can maintain its excellent photocatalytic performance after long-term environmental tests (>5000 h).

Furthermore, it has been observed that these photocatalysts exhibit exceptional photocatalytic efficiency, as they are capable of directly oxidizing organic substances by the action of h^+^ ions without the need for the production of intermediary reactive oxygen species derived from water or oxygen (such as ·OH or ·O_2_^−^). Directly harnessing holes for oxidation processes can streamline the steps involved in generating reactive oxygen species, effectively minimizing potential energy waste and the formation of reaction by-products. The utilization of hole-driven photocatalytic processes is crucial for enhancing the efficiency and selectivity of photocatalytic reactions. Meanwhile, in this photocatalytic process, the strong direct oxidation capacity of h^+^ makes Cs_2_PbI_2_Cl_2_-based photocatalysts exceptionally effective in treating difficult-to-degrade pollutants. Such applications amply warrant further exploration and research. In summary, this study presents a simple and attainable method to produce catalyst crystals based on Cs_2_PbI_2_Cl_2_, which are not quantum dots. The study also showcases the promising capabilities of these crystals as efficient and long-lasting photocatalysts driven by holes.

## Data Availability

The raw data supporting the conclusions of this article will be made available by the authors on request.

## Supplementary Materials

The following supporting information can be downloaded at: https://www.mdpi.com/article/10.3390/molecules29143249/s1. Figure S1: XRD patterns of CsPbBr_3_, Cs_2_PbI_2_Cl_2_, and Cs_2_PbI_2_Cl_2_/CsPbBr_3_ crystals; Figure S2: (a), (b) and (c) HRTEM images of Cs_2_PbI_2_Cl_2_/CsPbBr_3_ photocatalysts; Figure S3: (a) UV-vis absorption of organic solution without and with being catalyzed by CsPbBr_3_, Cs_2_PbI_2_Cl_2_ and Cs_2_PbI_2_Cl_2_/CsPbBr_3_ after 2 min (b) After 4 min (c) After 8 min (d) After 10 min; Figure S4: Photocatalytic performance tests of Cs_2_PbI_2_Cl_2_/CsPbBr_3_ and TiO_2_/CsPbBr_3_ photocatalysts; Figure S5: (a) EIS measurements of Cs_2_PbI_2_Cl_2_/CsPbBr_3_ and TiO_2_/CsPbBr_3_ photocatalysts, (b) transient photocurrent response tests of Cs_2_PbI_2_Cl_2_/CsPbBr_3_ and TiO_2_/CsPbBr_3_ photocatalysts; Figure S6: Accelerated aging testing of Cs_2_PbI_2_Cl_2_/CsPbBr_3_ and CsPbBr_3_ photocatalysts; Figure S7: Reaction rates of Cs_2_PbI_2_Cl_2_/CsPbBr_3_ and CsPbBr_3_-based photocatalytic processes fitted by using pseudo-first-order kinetics after accelerated aging tests.

## References

[1] Pastor E., Sachs M., Selim S., Durrant J.R., Bakulin A.A., Walsh A. (2022). Electronic Defects in Metal Oxide Photocatalysts. Nat. Rev. Mater..

[2] Gautam S., Agrawal H., Thakur M., Akbari A., Sharda H., Kaur R., Amini M. (2020). Metal Oxides and Metal Organic Frameworks for the Photocatalytic Degradation: A Review. J. Environ. Chem. Eng..

[3] Krishnan A., Swarnalal A., Das D., Krishnan M., Saji V.S., Shibli S.M.A. (2024). A Review on Transition Metal Oxides Based Photocatalysts for Degradation of Synthetic Organic Pollutants. J. Environ. Sci..

[4] Ryu J., Park D., Hahn B., Choi J., Yoon W., Kim K., Yun H. (2008). Photocatalytic TiO_2_ Thin Films by Aerosol-Deposition: From Micron-Sized Particles to Nano-Grained Thin Film at Room Temperature. Appl. Catal. B-Environ..

[5] Parrino F., Livraghi S., Giamello E., Ceccato R., Palmisano L. (2020). Role of Hydroxyl Superoxide and Nitrate Radicals on the Fate of Bromide Ions in Photocatalytic TiO_2_ Suspensions. ACS Catal..

[6] Zhu B., Sun J., Zhao Y., Zhang L., Yu J. (2023). Construction of 2D S-Scheme Heterojunction Photocatalyst. Adv. Mater..

[7] NREL Best Research Cell Efficiency Records. https://www.nrel.gov/pv/cell-efficiency.html.

[8] Feng J., Mak C.H., Yu L., Han B., Shen H., Santoso S.P., Yuan M., Li F., Song H., Colmenares J.C. (2024). Structural Modification Strategies Interfacial Charge-Carrier Dynamics and Solar Energy Conversion Applications of Organic-Inorganic Halide Perovskite Photocatalysts. Small Methods.

[9] Huang Y., Yu J., Wu Z., Li B., Li M. (2024). All-Inorganic Lead Halide Perovskites for Photocatalysis: A Review. RSC Adv..

[10] Mathuri A., Pal B., Pramanik M., Manna A., Mal P. (2024). Enhancing the Photocatalytic Efficiency and Stability of CsPbBr_3_ Nanocrystals for Visible-Light Driven Aerobic Diaryl Thio/Seleno Etherification. Catal. Sci. Technol..

[11] Song W., Chong K.C., Qi G., Xiao Y., Chen G., Li B., Tang Y., Zhang X., Yao Y., Lin Z. (2024). Unraveling the Transformation from Type-II to Z-Scheme in Perovskite-Based Heterostructures for Enhanced Photocatalytic CO_2_ Reduction. J. Am. Chem. Soc..

[12] Gao S., Wang B., Chen F., He G., Zhang T., Li L., Li J., Zhou Y., Feng B., Mei D. (2024). Confinement of CsPbBr_3_ Perovskite Nanocrystals into Extra-Large-Pore Zeolite for Efficient and Stable Photocatalytic Hydrogen Evolution. Angew. Chem. Int. Ed..

[13] Cai Y., Luo Q., Jiang Q., Liu X., Chen X., Liu J., Mao X., Qi J., Liang R., Qiu J. (2024). Hydrogen-Bonded Cocrystals Encapsulating CsPbBr_3_ Perovskite Nanocrystals with Enhancement of Charge Transport for Photocatalytic Reduction of Uranium. Small.

[14] Zhu X., Lin Y., San Martin J., Sun Y., Zhu D., Yan Y. (2019). Lead Halide Perovskites for Photocatalytic Organic Synthesis. Nat. Commun..

[15] Cardenas Morcoso D., Gualdron Reyes A.F., Ferreira Vitoreti A.B., Garcia Tecedor M., Yoon S.J., De La Fuente M.S., Mora Sero I., Gimenez S. (2019). Photocatalytic and Photoelectrochemical Degradation of Organic Compounds with All-Inorganic Metal Halide Perovskite Quantum Dots. J. Phys. Chem. Lett..

[16] Jiang Y., Chen H., Li J., Liao J., Zhang H., Wang X., Kuang D. (2020). Z-Scheme 2D/2D Heterojunction of CsPbBr_3_/Bi_2_WO_6_ for Improved Photocatalytic Co_2_ Reduction. Adv. Funct. Mater..

[17] Liu W., Liu J., Wang X., He J., Li Y., Liu Y. (2023). Synthesis of Asymmetrical CsPbBr_3_/TiO_2_ Nanocrystals with Enhanced Stability and Photocatalytic Properties. Catalysts.

[18] Xu Y., Yang M., Chen B., Wang X., Chen H., Kuang D., Su C. (2017). A CsPbBr_3_ Perovskite Quantum Dot/Graphene Oxide Composite for Photocatalytic CO_2_ Reduction. J. Am. Chem. Soc..

[19] Sanjayan C.G., Jyothi M.S., Balakrishna R.G. (2022). Stabilization of Cspbbr_3_ Quantum Dots for Photocatalysis Imaging and Optical Sensing in Water And Biological Medium: A Review. J. Mater. Chem. C.

[20] Rasool R.T., Ashraf G.A., Pasha M., Saleem M.F., Ghernaout D., Fadhali M.M., Guo H. (2023). Nanoscaled MnSnO_2_@CsPbBr_3_ Quantum Dots Heterostructure Photocatalyst as Efficient Organic Pollutants Degradation by Peroxymonosulfate; DFT Calculation. J. Mater. Sci. Technol..

[21] Jiang H., Liu M., Lian X., Zhu M., Zhang F. (2024). CsPbBr_3_ Quantum Dots Promoted Depolymerization of Oxidized Lignin via Photocatalytic Semi-Hydrogenation/Reduction Strategy. Angew. Chem. Int. Ed..

[22] Zhong F., Sheng J., Du C., He Y., Sun Y., Dong F. (2024). Ligand-Mediated Exciton Dissociation and Interparticle Energy Transfer on Cspbbr_3_ Perovskite Quantum Dots for Efficient CO_2_-To-CO Photoreduction. Sci. Bull..

[23] Yan D.D., Shi T.C., Zang Z.G., Zhou T.W., Liu Z.Z., Zhang Z.Y., Du J., Leng Y.X., Tang X.S. (2019). Ultrastable Cspbbr_3_ Perovskite Quantum Dot and Their Enhanced Amplified Spontaneous Emission by Surface Ligand Modification. Small.

[24] Chen L.C., Tien C.H., Tseng Z.L., Dong Y.S., Yang S.Y. (2019). Influence of Pmma on All-Inorganic Halide Perovskite CsPbBr_3_ Quantum Dots Combined with Polymer Matrix. Materials.

[25] Liu Y., Li Y.L., Hu X.D., Wei C.T., Xu B., Leng J., Miao H.B., Zeng H.B., Li X.M. (2023). Ligands for CsPbBr_3_ Perovskite Quantum Dots: The Stronger the Better?. Chem. Eng. J..

[26] Xu Y.Y., Niu P.J., Zhang L., Wen Z.Y., Cheng S., Lyu M., Zhu J. (2023). Tailoring Multifunctional Anions to Inhibit Methanol Absorption on A CsPbBr_3_ Quantum Dot Surface for Highly Efficient Semi-Transparent Photovoltaics. Nanoscale.

[27] Mathews N.R., Morales E.R., Cortés-Jacome M.A., Antonio J.A.T. (2009). TiO_2_ Thin Films—Influence of Annealing Temperature on Structural Optical and Photocatalytic Properties. Sol. Energy.

[28] Landes C., Burda C., Braun M., El-Sayed M.A. (2001). Photoluminescence of CdSe Nanoparticles in the Presence of a Hole Acceptor: N-Butylamine. J. Phys. Chem. B.

[29] Akasheh F., Karim M.R., Shao S. Dislocation Structure of Cu/Nu (100) Semi-Coherent Interface and Its Role in Lattice Dislocation Nucleation. Proceedings of the TMS 2015 144th Annual Meeting & Exhibition.

[30] Wang X., Zhong Y., Wang D., Sun L., Jiang B., Wang J. (2018). Effect of interfacial energy on microstructure of a directionally solidified Al_2_O_3_/YAG eutectic ceramic. J. Am. Ceram. Soc..

[31] Zhu Y., Xue J., Xu T., He G., Chen H. (2017). Enhanced Photocatalytic Activity of Magnetic Core–shell Fe_3_O_4_@Bi_2_O_3_–RGO Heterojunctions for Quinolone Antibiotics Degradation under Visible Light. J. Mater. Sci. Mater. Electron..

[32] Ye L., Liu J., Gong C., Tian L., Peng T., Zan L. (2012). Two Different Roles of Metallic Ag on Ag/AgX/BiOX (X = Cl, Br) Visible Light Photocatalysts: Surface Plasmon Resonance and Z-Scheme Bridge. ACS Catal..

