# Supplementary material for: Accessible New Non-Quantum Dot Cs2PbI2Cl2-Based Photocatalysts for Efficient Hole-Driven Photocatalytic Applications
Source: Molecules. 2024 Jul 9;29(14):3249. doi: 10.3390/molecules29143249 (PMC11278695; doi:10.3390/molecules29143249)
Supplement: Supplementary file 1 [file molecules-29-03249-s001.zip › molecules-3068690-supplementary.pdf]

Accessible New Non-Quantum Dot $Cs_2PbI_2Cl_2$-Based Photocatalysts for Efficient Hole-Driven Photocatalytic Applications

Xing Huang, Kuanxin Lv, Wenqiang Zhu, Zhenzhen Li * and Hang Zhao *

College of Metallurgy and Energy, North China University of Science and Technology, Tangshan 063210, China
* Correspondence: lizhenzhen@ncst.edu.cn (Z.L.); zhh1@ncst.edu.cn (H.Z.)

Experimental Section

Materials

All chemicals and reagents used in this experiment were purchased from suppliers without further purification. lead bromide ($PbBr_2$, 99.5%), cesium bromide (CsBr, 99.9%), cesium chloride (CsCl, 99.99%), lead iodide (PbI_2, 99.99%), dimethyl sulfoxide (DMSO, 99.9%), Rhodamine B (AR), Methanol (MT, 99.9%), Isopropyl alcohol (IPA, ≥99.9%), p-benzoquinone (p-BQ, 99%), N,N-dimethylformamide (DMF, 99.9%), silicone oil, ethanol (C_2H_6O, 99.7%), and titanium dioxide (particle size: 20 nm).

Preparation of $CsPbBr_3$: Adding 1.2 mmol of $PbBr_2$ and CsBr in equimolar ratio into 3 mL anhydrous DMSO, under an 80 °C oil bath, stirring continuously to dissolve solid particles and form a solution. The solution is then transferred into a preheated silicon oil solution at 110 °C to evaporate the DMSO, followed by continuous heating for 6 h until the solvent completely evaporates to yield $CsPbBr_3$ crystals.

Preparation of $Cs_2PbI_2Cl_2$: Add 0.6 mmol of CsCl and 0.3 mmol of PbI_2 powder to a 3 mL DMSO solution. To ensure complete dissolution of solid particles, heat the solution to 80 °C and stir continuously for 30 min. During this process, solid particles gradually dissolve, forming a homogeneous yellow solution. By slowly increasing the temperature of the solution to 140 °C through heating the silicon oil bath, continue heating for 5 h to obtain $Cs_2PbI_2Cl_2$ crystals.

Preparation of $CsPbBr_3/TiO_2$ heterostructure crystals: Using the same

high-temperature oil bath evaporation method, 20 mg of the prepared $CsPbBr_3$ crystals were weighed and placed in a beaker. 1 mL of DMSO was added, and the mixture was heated and stirred at 80 °C to dissolve. Then, 800 mg of TiO_2 powder was added, and the temperature was further increased to 140 °C while stirring until a paste-like consistency was obtained. The mixture was then dried in a vacuum at 90 °C for 12 h.

Preparation of $Cs_2PbI_2Cl_2/CsPbBr_3$ heterostructure crystals: First, add 0.6 mmol of CsCl and 0.3 mmol of PbI_2 powder to a 3 mL DMSO solution. Then, heat the mixed solution to 80 °C and stir continuously for 30 min. Next, add 0.2 mmol of pre-synthesized $CsPbBr_3$ crystals. Finally, raise the solution temperature to 140°C and continue heating for 5 h until completely dried to obtain the $Cs_2PbI_2Cl_2/CsPbBr_3$ heterostructure.

Photocatalytic Degradation of Rhodamine B

Dissolve 5 mg of Rhodamine B powder in 1.5 mL of ethanol and add hexane solvent to make a 100 mL solution. After ultrasonication for one minute to ensure uniform dispersion of Rhodamine B in the solution, add 6 mg powder of photocatalytic material (Among them, the concentration of $Cs_2PbI_2Cl_2$ is 0.12 mg/mL, and the concentration of $CsPbBr_3$ is 0.08 mg/mL) to 30 mL of the Rhodamine B solution and proceed with the photocatalytic process under an AM 1.5G xenon lamp.

(Photo)electrochemistry Characterization

The (photo)electrochemical characterization was performed using a CHI 760E electrochemical workstation. Measurements were conducted in a three-electrode setup with a platinum electrode as the working electrode, an Ag/AgCl electrode as the reference electrode, and the sample electrode as the working electrode. For photocurrent measurements, a xenon lamp (300 W) was used with a light-on/off interval of 20 s (20 s light-on, 20 s light-off), and the cycle was repeated at least four times. EIS measurements were carried out in a 0.5 M Na_2SO_4 electrolyte solution, within a frequency range of 0.1 Hz to 100 K Hz, the amplitude is 5 mV, Set bias voltage to −0.3 V, NHE by the formula of $E_{NHE} = E_{Ag/AgCl} + 0.1989$ V, under xenon

lamp illumination.

Material Characterization

X-ray diffraction (XRD) analysis was performed using a diffractometer (Rigaku Ultima IV, Japan) with Cu Kα radiation (wavelength of 1.5418 Å, voltage of 40 kV, and current of 40 mA). Scanning electron microscopy (SEM, Zeiss Merlin Compact, Germany) was used to characterize the morphology and grain size of the powder samples. Transmission electron microscopy (TEM, Thermo Scientific Talos F200X G2, USA) was employed for high-resolution analysis of the crystal structure. The absorbance of the sample solution was recorded using a UV-visible-near-infrared spectrophotometer (Hitachi U4150, Japan), with $BaSO_4$ powder as a reference in the range of 250-800 nm for optical property measurements of the catalyst powder. X-ray photoelectron spectroscopy (XPS) was used to determine the energy band structure of the materials. Electrochemical impedance spectroscopy (EIS) and photocurrent density measurements of the materials were performed using a CHI 760E electrochemical workstation. Electron paramagnetic resonance (EPR) spectroscopy was conducted on an EPR spectrometer (Bruker EMX plus-6/1) using 2,2,6,6-tetramethylpiperidinooxy (TEMPO) as a spin trapping agent to capture the active radicals generated during the photocatalytic process. MT, IPA, and p-BQ were added to the catalytic solution to selectively quench h^+, $\cdot OH$, and $\cdot O_2^-$. Samples were taken every two mins to measure the remaining amount of Rhodamine B, indicating the extent of the influence of active radicals on the reaction.

Supporting Figures

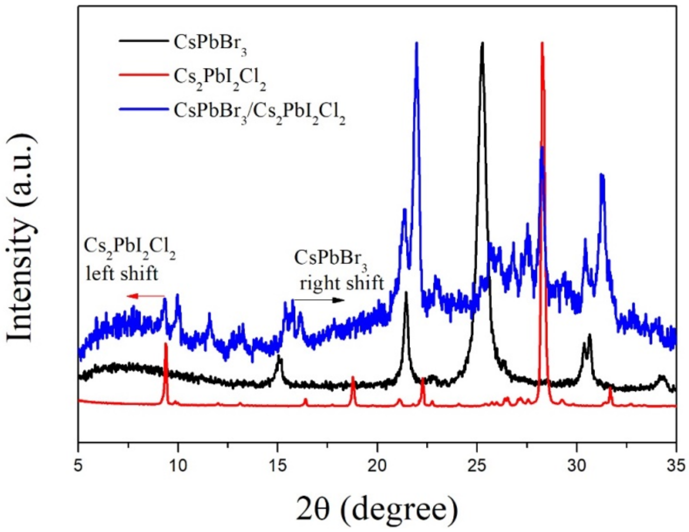

Figure S1. XRD patterns of $CsPbBr_3$, $Cs_2PbI_2Cl_2$, and $Cs_2PbI_2Cl_2/CsPbBr_3$ crystals.

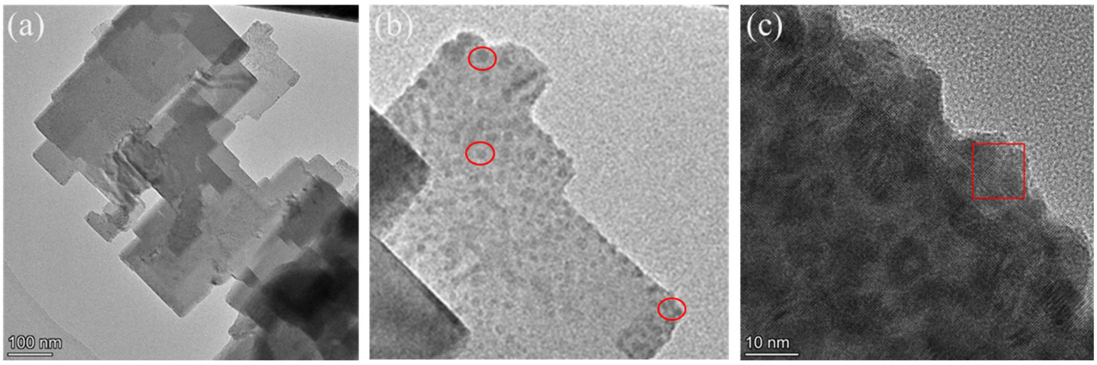

Figure S2. (**a**–**c**) HRTEM images of $Cs_2PbI_2Cl_2/CsPbBr_3$ photocatalysts.

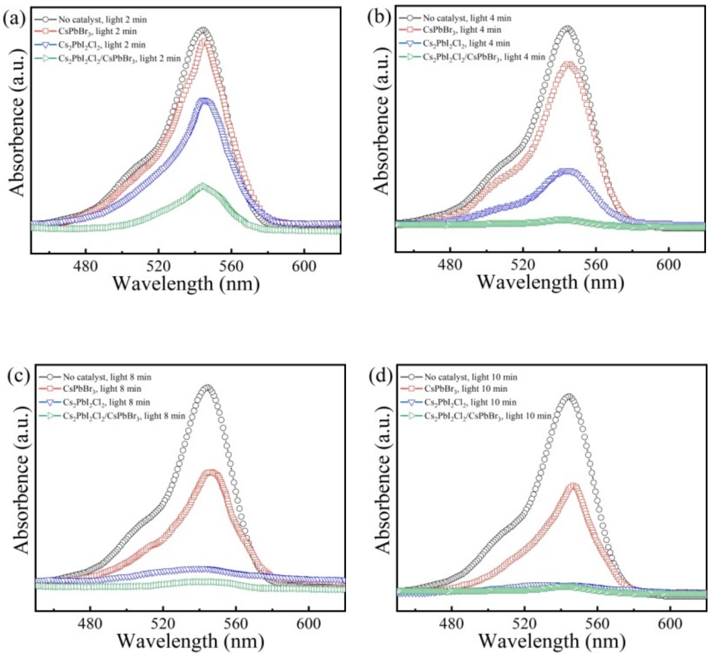

Figure S3. (**a**) UV-vis absorption of organic solution without and with being catalyzed by $CsPbBr_3$, $Cs_2PbI_2Cl_2$ and $Cs_2PbI_2Cl_2/CsPbBr_3$ after 2 min. (**b**) After 4 min. (**c**) After 8 min. (**d**) After 10 min.

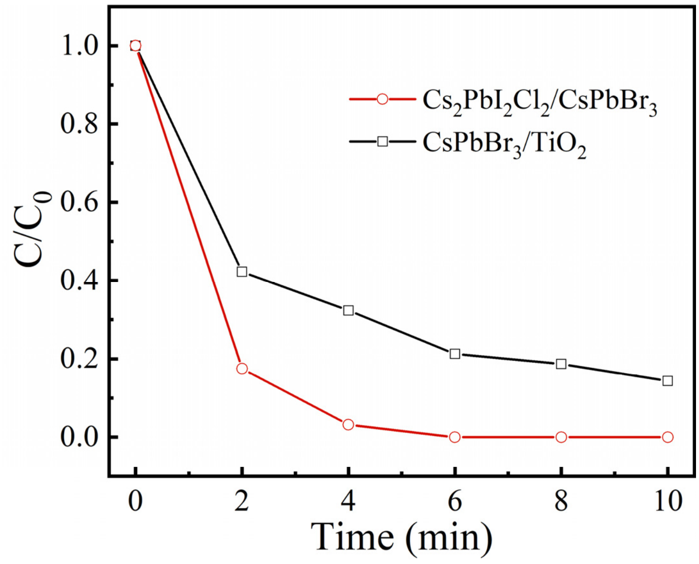

Figure S4. Photocatalytic performance tests of $Cs_2PbI_2Cl_2/CsPbBr_3$ and $TiO_2/CsPbBr_3$ photocatalysts.

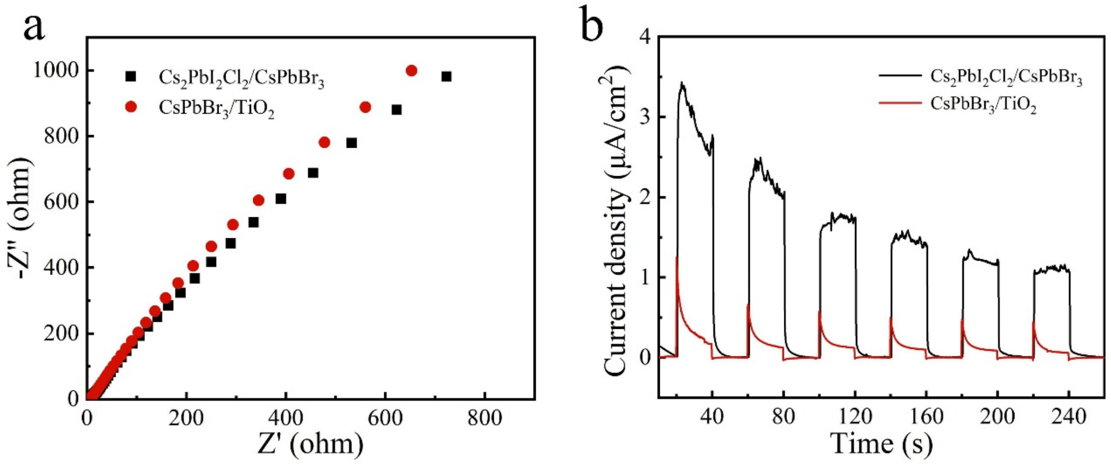

Figure S5. (**a**) EIS measurements of $Cs_2PbI_2Cl_2/CsPbBr_3$ and $TiO_2/CsPbBr_3$ photocatalysts, (**b**) transient photocurrent response tests of $Cs_2PbI_2Cl_2/CsPbBr_3$ and $TiO_2/CsPbBr_3$ photocatalysts.

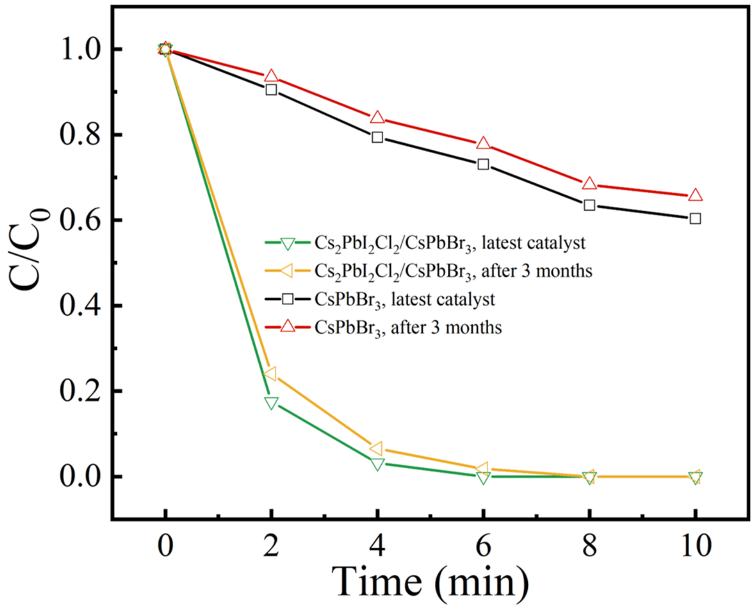

Figure S6. Accelerated aging testing of $Cs_2PbI_2Cl_2/CsPbBr_3$ and $CsPbBr_3$ photocatalysts.

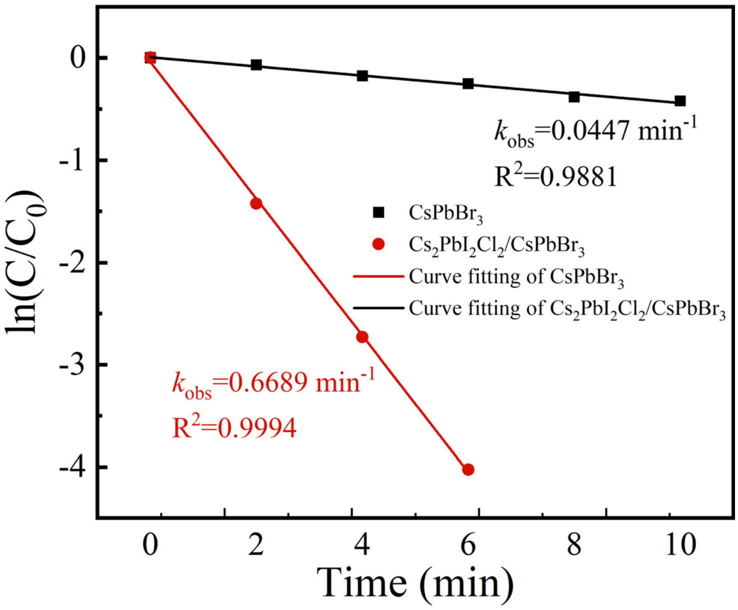

Figure S7. Reaction rates of $Cs_2PbI_2Cl_2/CsPbBr_3$ and $CsPbBr_3$-based photocatalytic processes fitted by using pseudo-first-order kinetics after accelerated aging tests.
